# Ten Broad Spectrum Resistances to Downy Mildew Physically Mapped on the Sunflower Genome

**DOI:** 10.3389/fpls.2018.01780

**Published:** 2018-12-04

**Authors:** Yann Pecrix, Charlotte Penouilh-Suzette, Stéphane Muños, Felicity Vear, Laurence Godiard

**Affiliations:** ^1^Laboratoire des Interactions Plantes Microorganismes, INRA, CNRS, Université de Toulouse, Castanet-Tolosan, France; ^2^INRA, Génétique, Diversité, Ecophysiologie des Céréales, UMR 1095, Clermont-Ferrand, France

**Keywords:** *Helianthus annuus*, Plasmopara halstedii, resistance gene (R gene), SNP array, oomycete, RXLR effector, hypersensitive response (HR), crop protection

## Abstract

Resistance to downy mildew (*Plasmopara halstedii*) in sunflower (*Helianthus annuus* L.) is conferred by major resistance genes, denoted *Pl.* Twenty-two *Pl* genes have been identified and genetically mapped so far. However, over the past 50 years, wide-scale presence of only a few of them in sunflower crops led to the appearance of new, more virulent pathotypes (races) so it is important for sunflower varieties to carry as wide a range of resistance genes as possible. We analyzed phenotypically 12 novel resistant sources discovered in breeding pools derived from two wild *Helianthus* species and in eight wild *H. annuus* ecotypes. All were effective against at least 16 downy mildew pathotypes. We mapped their resistance genes on the sunflower reference genome of 3,600 Mb, in intervals that varied from 75 Kb to 32 Mb using an AXIOM^®^ genotyping array of 49,449 SNP. Ten probably new genes were identified according to resistance spectrum, map position, hypersensitive response to the transient expression of a *P. halstedii* RXLR effector, or the ecotype/species from which they originated. The resistance source HAS6 was found to carry the first downy mildew resistance gene mapped on chromosome 11, whereas the other resistances were positioned on chromosomes 1, 2, 4, and 13 carrying already published *Pl* genes that we also mapped physically on the same reference genome. The new genes were designated *Pl_23_–Pl_32_* according to the current nomenclature. However, since sunflower downy mildew resistance genes have not yet been sequenced, rules for designation are discussed. This is the first large scale physical mapping of both 10 new and 10 already reported downy mildew resistance genes in sunflower.

## Introduction

Downy mildew of sunflower, caused by *Plasmopara halstedii* can occur in most countries where sunflowers (*Helianthus annuus* L.) are grown. It is an obligate parasitic oomycete belonging to the Peronosporales showing physiological races or pathotypes (Gascuel et al., 2015) which interact with sunflower genotypes according to the resistance genes they contain. More than 20 pathotypes have been described worldwide (Viranyi et al., 2015) and 22 major resistance genes (denoted *Pl*) have been identified in sunflower (Qi et al., 2016b). Sunflower was domesticated by Native Americans from wild *H. annuus* but the first large scale developments of the crop were in Russia between 1900 and 1950. *P. halstedii* also originated in North America (Leppik, 1962) but the first important disease attacks occurred in Russia (Pustovoit, 1966) when sunflower became an important crop. In the period 1960–70, G.V. Pustovoit introduced resistance from *H. tuberosus* into cultivated sunflower and bred open pollinated varieties (OPV) Progress and Novinka which were resistant to downy mildew in Russia. When research on sunflower started in Canada, accidental crosses with wild *H. annuus* in 1962–1963 showed that resistance was available in this weedy species (Putt, 1964). In Western Europe, where the climate is favorable for downy mildew, the genes *Pl*_1_ and *Pl_2_*, from wild *H. annuus*, were rapidly incorporated into hybrid varieties and it became clear that the pathotype prevalent in Europe (100) was not the same as that in the United States (300) (Zimmer and Kinman, 1972). Between 1978 and 1988, nearly all varieties carried these genes and the appearance, first in the United States and then in Europe, of new pathotypes not controlled by these genes (700, 703, 710) (Gulya et al., 1991) may well have been caused by the selection pressure applied on *P. halstedii* as well as by accidental downy mildew introductions in sunflower seed (Ahmed et al., 2012). Progress and Novinka and also some ecotypes of wild *H. annuus* and other annual *Helianthus* spp. (Miller and Gulya, 1988) provided effective resistance. However, in France, rapid appearance of further new pathotypes (304, 314, 334) provided confirmation that this was favored if all widely grown varieties carried the same resistance genes and that it was important to make a search for additional genes (Tourvieille de Labrouhe, 2003).

Studies have been made of quantitative, perhaps pathotype-non-specific, resistance (Tourvieille de Labrouhe et al., 2008; Vear et al., 2008b; Vincourt et al., 2012) but this is not easy to use in breeding, so control of downy mildew in sunflower still depends largely on major resistance genes. It is thus most important to find a wide range of genes giving resistance to the most prevalent pathotypes in areas where sunflower is an important crop.

Several countries have developed collections of wild *H. annuus* and other *Helianthus* species, and research for new downy mildew resistance genes has been made in crosses of this material with cultivated sunflower. The genes introduced into cultivated sunflower up to 2008 were summarized by Vear et al. (2008a). Since then, *Pl_14_* (Bachlava et al., 2011) and *Pl_15_* (Bertero de Romano et al., 2010) were identified in Argentina from local OPV and *Pl_13_* (Mulpuri et al., 2009) and *Pl_16_* (Liu et al., 2012) in the United States from similar origins; *Pl_17_* and *Pl_19_* originated from wild *H. annuus* (Qi et al., 2015; Zhang et al., 2017) and *Pl_18_* and *Pl_20_* were introduced from *H. argophyllus* (Qi et al., 2016a; Ma et al., 2017). *Pl_21_*, identified in many restorer genotypes, probably originated from the same wild *H. annuus* as *Pl*_2_ (Vincourt et al., 2012). *Pl_2_*_2_, obtained from the OPV Novinka, should be from *H. tuberosus* but could be from *H. argophyllus* according to its mapping position on the reference genome (Pecrix et al., 2018).

To determine whether different resistant accessions contain different genes, the classic method is to study phenotypic reactions of progenies from crosses between resistant lines. However, additional genotypic analyses and fine genetic mapping are necessary to obtain more precise conclusions. Most of the downy mildew resistance genes identified since 1972 have been localized on molecular maps using either RFLP (*Pl*_1_, *Pl*_2_, *Pl*_5_, *Pl*_6/7_, *Pl*_8_), SSR (simple sequence repeats) (*Pl_ARG_*, *Pl*_13_, *Pl*_15_, *Pl*_18_, *Pl*_19_, *Pl*_21_) and more recently SNP (single nucleotide polymorphisms) (*Pl*_17_, *Pl*_20_, *Pl*_22_). *Pl*_22_ was the first resistance gene to be physically mapped to a region of 1.7 Mb of chromosome 13 (Pecrix et al., 2018). Some genes have been difficult to localize; for example *Pl*_13_ (Mulpuri et al., 2009) and *Pl*_22_ could not be localized by Bulk Segregant Analyses with RFLP and SSR in 2000–2005. In addition, several *Pl* genes are clustered in complex genomic regions on chromosomes 1, 2, 4, 8, and 13 carrying many resistance gene candidates (RGCs; Radwan et al., 2003, 2008) and showing duplications (Bachlava et al., 2011). It is therefore not easy to localize precisely the different genes relative to each other, especially if different polymorphic markers are used to map them. Physical mapping of both known and novel resistance *Pl* genes on the reference genome sequence is therefore a major progress and should be an asset for breeders.

Oomycete pathogens produce virulence protein factors called effectors, secreted and translocated in plants to facilitate colonization (Fawke et al., 2015). Mestre et al. (2016) showed that *P. halstedii* expressed effectors during sunflower infection and overexpression of some cloned effectors induced hypersensitive reactions (HRs) in resistant sunflower lines and not in near-isogenic susceptible lines (Gascuel et al., 2016). Specific recognition of effectors acting as avirulent factors has been used as a reliable marker of resistance genes in potato (Vleeshouwers et al., 2008) and in sunflower (Gascuel et al., 2016; Pecrix et al., 2018). We used this property to distinguish some closely linked resistances.

This paper reports genetic mapping of the broad spectrum resistance genes in 12 sunflower lines developed from crosses of cultivated and wild *Helianthus* spp., and their physical mapping on the sunflower reference genome using an AXIOM^®^ SNP genotyping array. Ten putatively new downy mildew resistance genes were located on the sunflower genome sequence together with 10 already published *Pl* genes mapped on the same chromosomes. The question of what constitutes a new resistance gene is discussed.

## Materials and Methods

### Sunflower Genotypes and Crosses Made

In 2005–2006, downy mildew resistance was identified in interspecific pools and progenies from crosses of cultivated and wild *H. annuus* developed using the INRA collection. Inbred lines were developed from progenies showing resistance to both pathotypes 710 and 304 and were found to give resistance to all or most French pathotypes (Vear et al., 2008a). The inbred lines carrying known downy mildew resistance genes and these new sources of resistance are listed in Table 1. HIS or INTER denotes origins from interspecific pools, HAS denotes hybridization with wild *H. annuus*. The line IDAHO was bred from USDA material from which HA458 was also developed (Hulke et al., 2010). It was crossed with PMI3 in 2006 to study both resistance origins. Table 2 presents resistances of all the new lines to 15 French pathotypes of *P. halstedii* and a Spanish pathotype 330 not present in France.

**Table 1:** Pedigrees of known and new inbred sunflower lines with downy mildew resistance.

Resistant line	INRA code	Pedigree	Downy mildew resistance gene	Source of resistance	Breeder
**New downy mildew resistance sources**					
HIS33	UTQ	INRA interspecific breeding pool *H. resinosus 1451*		*H. resinosus 1451*	INRA
INTER35	UUQ	INRA interspecific breeding pool *H. resinosus 1323*		*H. resinosus 1323*	INRA
HAS62	HAS62	(VKQ × (OEG × (*H. annuu*s Utah 775RT1A11)))		*H. annuus Utah 775*	INRA
HAS40	HBQ	(*(H. annuus* Texas 650 × RT1A11) × OEK)		*H. annuus Texas650*	INRA
HAS103	HAS103	(OEG ×*(H. annuus* Kansas 999 × 90R19))		*H. annuus Kansas 999*	INRA
IDAHO	IDAHO	(HA434 ×*H. annuus* Idaho PI468435)F4		*H. annuus* Idaho	USDA/INRA
HIS32	HIS32	(VKQ × INRA interspecific breeding pool *H. tomentosus 1450)*		*H. tomentosus 1450*	INRA
HIS36	HIS36	*INRA interspecific breeding pool H. tomentosus*		*H. tomentosus*	INRA
HAS85	HEQ	(OW ×*(H. annuus* Wyoming 963 × RT1A11))		*H. annuus Wyoming 963*	INRA
HAS6	HAQ	(OW ×*(H. annuus* Wyoming 358 × RT1A11))		*H. annuus Wyoming 358*	INRA
HAS42	HCQ	((*H. annuus* Texas 651RT1A11) × OEU)		*H. annuus Texas 651*	INRA
HAS54	HAS54	(OZ × (*H. annuus* Oklahoma 662 × 90R19))		*H. annuus Oklahoma 662*	INRA
**Lines with known downy mildew resistance genes**					
PAA1	PAA1	(*AR22.A.A.B.D.D* (*from hybrid with H. argophyllus*) × PBP1)	*Pl_ARG_*	AR22 – *H. argophyllus*	INRA
0QP7	0QP7	(PAA1 × RHA345)	*Pl_ARG_*	PAA1 – *H. argophyllus*	INRA
0QP8	0QP8	(PAA1 × RHA345)	*Pl_ARG_*	PAA1 – *H. argophyllus*	INRA
RHA419	RHA419	(RHA373 ×*H. argophyllus* 1575-2)	*Pl_ARG_*	*H. argophyllus 1575*	USDA
HA458	COQ	(HA434 ×*H. annuus* Idaho PI468435)	*Pl_*17*_*	*H. annuus* Idaho	USDA
VKQ	VKQ	(H52 × YDQ (HA335 *H. annuus* Texas))	*Pl_*6*_*	HA335 – *H. annuus*	INRA
XRQ	XRQ	(HA89 × Progress 1816)	*Pl_*5*_*	Progress – *H. tuberosus*	INRA
HA460	U8Q	(HA434 × RHA340)	*Pl_*8*_*	RHA340 – *H. argophyllus*	USDA

*Inbred lines of cultivated sunflower in the pedigrees of new resistance sources were all bred by INRA (Institut National de la Recherche Agronomique, France). USDA, United States Department of Agriculture*.

**Table 2:** Reaction of sunflower resistance sources to 15 *Plasmopara halstedii* pathotypes found in France and *P. halstedii* pathotype 330 from Spain.

	16 *P. halstedii pathotypes*															
Sunflower genotype	100	300	304-10^a^	304-30^a^	307	314	*330*	334	700	703	704	707	710	714	730	774
HIS33	R	R	R	R	R	R	R	R	R	R	R	R	R	R	R	R
INTER35	R	R	R	R	R	R	R	R	R	R	R	R	R	R	R	R
HAS62	R	R	R	R	R	R	R	R	R	R	R	R	R	R	R	R
HAS40	R	R	R	R	R	R	R	R	R	R	R	R	R	R	R	R
HAS103	R	R	R	R	R	R	Rll	R	R	R	R	R	R	R	R	Rll
HAS85	R	R	R	R	R	R	R	R	R	R	R	R	R	R	R	R
HIS32	R	R	R	R	R	R	R	R	R	R	R	R	R	R	R	R
HIS36	R	R	R	R	R	R	R	R	R	R	R	R	R	R	R	R
IDAHO	R	R	R	R	R	R	R	R	R	R	R	R	R	R	R	R
HAS6	R	R	R	R	R	R	R	R	R	R	R	R	R	R	R	R
HAS42	R	R	R	R	R	R	R	R	R	R	R	R	R	R	R	R
HAS54	R	R	R	R	R	R	R	R	R	R	R	R	R	R	R	R

*Plants with no sporulation (R) or with sporulation only on cotyledons (RII) were considered as resistant; those with sporulation on cotyledons and leaves were noted as susceptible (S). ^*a*^Samples of 304 collected in 2000 and 2002, named according to the proposition of Tourvieille de Labrouhe et al. (2012) using additional differentials*.

To determine the number of genes controlling resistance in these new lines and, at the same time whether some are the same (or at least clustered) or whether they are inherited independently, the new lines were crossed firstly with lines representing the resistance genes clusters on chromosomes 1, 8, and 13 together with COQ (HA458) whose gene had not yet been mapped (now located on chromosome 4 (Qi et al., 2015), and then between each other, for most of the possible combinations (Supplementary Table 1). With knowledge of the origin of IDAHO, this line was not used to produce test crosses. The F1 hybrids were crossed with downy mildew susceptible lines to obtain test cross progenies which were tested for their downy mildew resistance to determine segregations indicating allelism or independent inheritance.

In addition, to prepare for molecular mapping, the new lines were crossed with lines susceptible to at least some downy mildew pathotypes. F1 and F2 generations were selfed in order to develop F3 progenies which were phenotyped to determine the genotype of the F2 plants from which they were derived (Table 3).

**Table 3:** Downy mildew reactions for F3 progenies from crosses of new resistance sources with lines susceptible to the pathotypes tested.

Cross	Downy mildew pathotype	Number of F3 progenies	Results	Theoretical segregation		X^2^	Notes
HIS33 × OPC3	710	101	24RR : 53SEG : 24SS	1 gene	1RR : 2SEG : 1SS	0.207ns	
PAS5 × lNTER35	710	165	24RR : 96SEG : 45SS	1 gene	1RR : 2SEG : 1SS	9.65^∗∗^	Many RII type plants
VKQ × HAS62	334	90	44RR : 37SEG : 9SS	2 genes	7RR : 8SEG : 1SS	2.18ns	
VKQ × HAS62	714	43	12RR : 22SEG : 9SS	1 gene	1RR : 2SEG : 1SS	0.442ns	
OEH × HAS40	710	141	34RR : 74SEG : 33SS	1 gene	1RR : 2SEG : 1SS	0.096ns	
VKQ+XRQ × HAS103	334	87	30RR : 39SEG : 18SS	1 gene	1RR : 2SEG : 1SS	4.24ns	
PMI3 × IDAHO	710	177	51RR : 81SEG : 45SS	1 gene	1RR : 2SEG : 1SS	1.678ns	
VKQ × HIS32	334	117	50RR : 59SEG : 8SS	2 genes	7RR : 8SEG : 1SS	0.096ns	
VKQ × HAS32	714	95	19RR : 60SEG : 16SS	1 gene	1RR : 2SEG : 1SS	6.77^∗∗a^	
VKQHIS36	334	72	17RR : 33SEG : 22SS	1 gene	1RR : 2SEG : 1SS	1.194ns	
Dl × HAS85	710	136	37RR : 70SEG : 29SS	1 gene	1RR : 2SEG : 1SS	1.96ns	
HAS6 × OPC2	334	130	38RR : 63SEG : 29SS	1 gene	1RR : 2SEG : 1SS	0.955ns	
HAS42 × PSM7	710	148	33RR : 82SEG : 33SS	1 gene	1RR : 2SEG : 1SS	1.73ns	
VKQ × HAS54	334	95	35RR : 53SEG : 7SS	2 genes	7RR : 8SEG : 1SS	1.283ns	
VKQ × HAS54	714	41	12RR : 18SEG : 11SS	1 gene	1RR : 2SEG : 1SS	0.659ns	

*RR, homozygous resistant; SEG, progeny segregating for resistance; SS, homozygous susceptible. ^*a*^If 4 of the 60 progenies noted SEG were in fact SS, the X^*2*^ would not be significant. ^∗∗^: significantly different P > 0.01; ns: not significantly different*.

### Downy Mildew Resistance Tests

Classic seed immersion downy mildew tests (Mouzeyar et al., 1993) were used to phenotype both test cross and F3 progenies. These tests were made in confined growth chambers in agreement with European quarantine regulations. Pathotypes (710, 304, 334, 714) were among those known to be present in France, and chosen to permit determination of the presence or absence of the resistance genes studied. As in previous studies (Vear et al., 1997), plants with no sporulation (R) or with sporulation only on cotyledons (RII) were counted as resistant; those with sporulation on cotyledons and leaves, whether profuse (S) or rare (SII) were considered as susceptible.

For each test cross progeny, the number of plants tested varied according to the number of seed available. All the results were summed, such that the total number of plants tested with one pathotype for one progeny varied between 91 and 763, except for one progeny with 51 plants, which showed no segregation for resistance (all numbers of plants tested are given in Supplementary Table 2). Chi square tests were made for segregation patterns: either no segregation (same gene or closely linked genes), 3R:1S (two independent genes) or 7R:1S (three independent genes).

For each F3 progeny, 20–25 seedlings were observed and, 72 to 177 F3 progenies for each resistance origin were tested using one pathotype and between 41 and 95 when tests were made with a second pathotype (Table 3). Chi-square tests were made for segregation patterns for one gene (1RR : 2SEG : 1SS) or for two genes (7RR : 8SEG :1SS). Progenies chosen for molecular analyses were preferentially those homozygous for resistance or susceptibility.

### Transient Expression Assays of a Cloned *P. halstedii* RXLR Effector

A construct consisting of the *P. halstedii* PhRXLR-C01 RXLR-type effector fused to YFP (Yellow Fluorescent protein) under the control of the 35S promoter was infiltrated in sunflower leaves of entire plants in *Agrobacterium*-mediated transient expression experiments, as previously described (Gascuel et al., 2016). The construct consisting of YFP under the control of the 35S promoter was used as an infiltration negative control. Two leaves of 12 plants were tested in two independent experiments and scored on a 0 to 5 cell death index scale (Gascuel et al., 2016). The triggering of an hypersensitive response (scores of the infiltrated leaf areas of 4 to 5) was indicative of the recognition of the effector by the resistance gene carried by the plant (Gascuel et al., 2016).

### Genotyping Procedures

From the phenotype segregation patterns, the numbers of F3 progenies chosen for genotyping each resistance source varied from 27 to 72, according to apparent similarity with another line or novelty, and to number of seeds available (Supplementary Table 3). Preference was given to progenies showing homozygous resistance or susceptibility. Leaf disks cut from eight seedlings, raised in a greenhouse, for each progeny, were bulked for DNA extraction. Extraction and hybridization experiments were conducted by the Gentyane platform (Plateforme Gentyane, UMR INRA/UBP 1095 Génétique Diversité et Ecophysiologie des Céréales, 5 chemin de Beaulieu – Clermont-Ferrand, France) on a GeneTitan^®^ (Affymetrix) according to the manufacturer’s instructions. Genotyping was performed using a 50K SNP AXIOM^®^ array (Mangin et al., 2017). Marker quality control and allele calling was carried out using the Axiom Analysis Suite software (v1.1.1.66 Affymetrix^1^), following the Best Practices Genotyping Workflow. Samples with a dish quality control (DQC) value ≥ 0.9 and QC call rate ≥ 0.90 thresholds were considered to have passed the quality control assessment. SNP quality control parameters and thresholds were defined as follows: polyploid species-type; cr-cutoff ≥ 90; fld-cutoff ≥ 3.6; het-so-cutoff ≥ -0.3; het-so-otv-cutoff ≥ -0.3; hom-ro-1-cutoff ≥ 0.6; hom-ro-2-cutoff ≥ 0.3; hom-ro-3-cutoff ≥ -0.9. For mapping, only PolyHighResolution SNPs considered to be reliable and informative were retained. A final SNP filter focused on genotype data of parental lines was applied: for either or both parents, markers that failed SNP calling, or were genotyped as heterozygous, or showed divergences of allele calling between technical replicates were discarded from the analysis.

### Mapping Procedures

The genetic maps were constructed using CarthaGène v1.2.3 software (de Givry et al., 2005). Markers were merged with the *mrkmerges* function and linkage groups were obtained with the *group 0.3 3* function for all mapping populations except for HAS6xOPC2 (*group 0.5 3*). The genetic distances between markers were calculated using the *lkh 1 -1* function and the genetic maps were obtained with the *bestprintd* function. Published linked markers of *Pl* genes were positioned on the sequenced sunflower genome (INRA inbred line XRQ; Badouin et al., 2017) using BLAST^2^ and the genetic and physical maps were drawn using Mapchart 2.3 software (Voorrips, 2002).

## Results

### Distinction of Downy Mildew Resistance Genes by Phenotypic Studies

Supplementary Table 2 presents the results of downy mildew resistance tests on all the test crosses, ordered according to the chromosome on which the downy mildew resistance has been mapped (as described below). The X^2^ were calculated for expected segregations in the case of two or three independent genes (3R:1S or 7R:1S), linkage was not considered. Ninety four of the 122 progeny segregations agreed with those expected (non-significant X^2^). In some cases, SII or RII plants could have caused some misinterpretation. Pathotype 710 was used for the majority of tests and gave the highest percentage of rejection of the null hypothesis (significant X^2^): 33% compared with 27% for pathotype 304 and 12% for pathotype 334. Among the lines used in the crosses, COQ (HA458) and HAS40 gave the largest numbers of significant X^2^, but in the case of HAS40, there were more segregations with unexpectedly low numbers of susceptible plants than those with excess susceptible plants. All X^2^ agreed with the hypotheses proposed for the line HIS32. From these results, the resistances of HIS33, INTER35 and HAS62 showed linkage to *Pl_ARG_* on chromosome 1, those of IDAHO, HIS32, HIS36 and HAS85 linkage to *Pl_*17*_* on chromosome 4 and those of HAS42 and HAS54 linkage to *Pl_*5*_* on chromosome 13, whereas resistances of HAS103 and HAS6 appeared independent of all the known *Pl* genes tested. It was only for HAS40 that no conclusion could be drawn; it showed few susceptible plants in crosses with *Pl_ARG_* but also in crosses involving *Pl_*17*_*.

Table 3 presents a summary of the reactions of F3 progenies. Tests were first made with pathotype 710, or pathotype 334 if the “susceptible” parent was VKQ, carrying *Pl_*6*_* conferring resistance to pathotype 710. Three lines, HAS62, HIS32, and HAS54 were found to show segregations indicating the presence of two genes for resistance to pathotype 334 although *Pl_*6*_* is not effective against this pathotype. The three lines are from quite different origins, but they appear to contain a *Pl_*2*_*-type gene in addition to that giving resistance to a large number of pathotypes. When tested with pathotype 714, the progenies showed segregations agreeing with the hypothesis for a single dominant gene. These segregations were used to define the resistance genotype of each F2 plant used for mapping.

**FIGURE 1:**
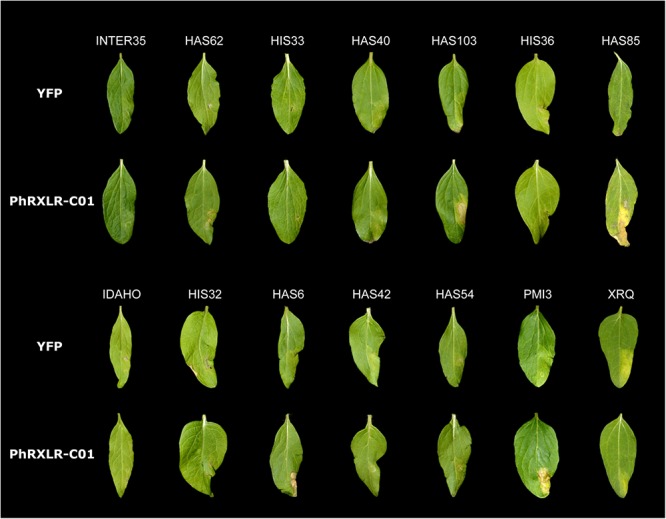
Recognition of *Plasmopara halstedii* PhRXLR-C01 effector in resistant sunflower genotypes by HR-like responses. Phenotypes observed in sunflower leaves of the 12 new resistant genotypes, after *Agrobacterium*-transient expression of the *P. halstedii* effector PhRXLR-C01 fused to YFP, or the YFP control, both driven by the 35S promoter. PMI3 (*Pl_*22*_*) and XRQ (*Pl_*5*_*) were used as positive and negative control lines to PhRXLR-C01 HR response, respectively. Pictures were taken 11 days post-infiltration (for more details see Gascuel et al., 2016). Sunflower leaf size varied from 5 to 6 cm long and 2 to 3 cm wide, depending on the tested line.

Transient expression assays with the PhRXLR-C01 effector of *P. halstedii* showed that only three of the 12 resistant lines responded by an HR: HAS103, HAS6 and HAS85, as well as the positive control line PMI3 (Pecrix et al., 2018) (Figure 1). All the other resistant lines showed no HR to the infiltration of the PhRXLR-C01 effector, suggesting that the resistances they carry do not recognize this specific effector. XRQ showed a discoloration phenotype with both YFP negative control and PhRXLR-C01 constructs, suggesting a stress response to the Agroinfiltration process in this ecotype but no specific recognition of the *P. halstedii* effector.

### Physical Mapping of the Resistances With SNP Markers on XRQ Reference Genome

The mapping interval of a resistance gene is delimited by the two closest markers, one on each side, revealing one recombination event between the resistance gene and the marker. This region may contain several markers co-segregating completely with resistance in the progeny tested. Supplementary Table 3 presents the numbers of polymorphic markers retained per chromosome and those used to map the resistance genes. For seven susceptible × resistant line progenies, a small number of SNP markers (from 0 to 10 for HAS42) co-segregated with resistance which was located in a physical interval of 75 to 4,222 Kb. Four progenies, on chromosomes 1 and 13 (HAS54), showed between 50 and 100 markers co-segregating with resistance, and larger interval sizes from 8.6 to 32 Mb (Supplementary Tables 3, 4). Mapping was not precise for the gene in the line HAS103 since we found 219 SNP markers of chromosome 2 (on a total of 345) co-segregating with resistance, and a physical interval for the resistance of 114 Mb.

#### Four New Resistances Map to Chromosome 1

**FIGURE 2:**
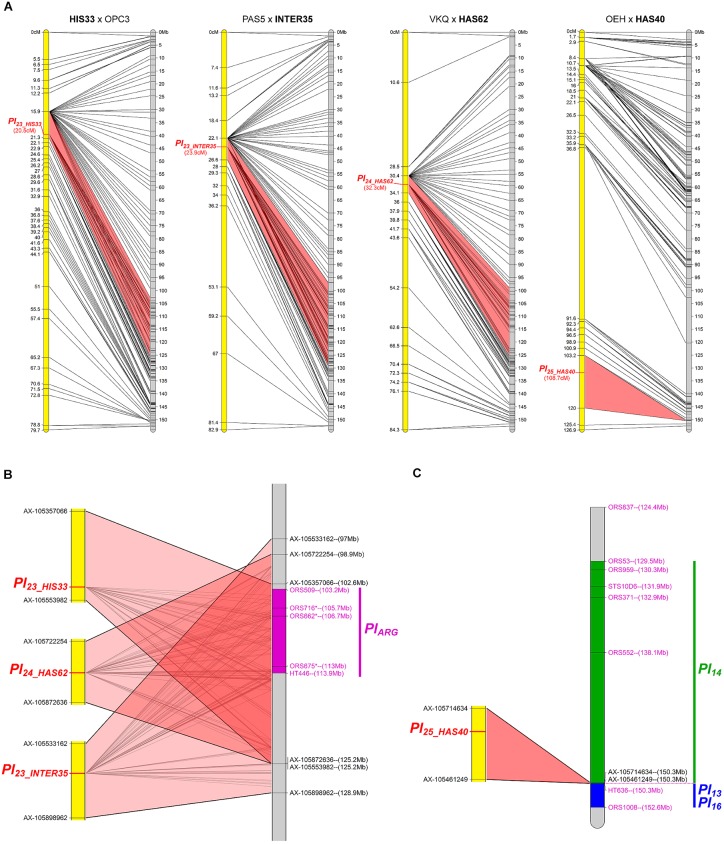
Four downy mildew resistances map to chromosome 1 of sunflower. **(A)** Genetic (yellow) and physical (gray) maps of the *Pl_*23_HIS33*_, Pl_*23_INTER35*_*, *Pl_*24_HAS62*_*, and *Pl_*25_HAS40*_* resistance genes on chromosome 1. The cross used to map the resistance is indicated on top of each map, with the resistant parent in bold. Only the non-redundant SNP markers are indicated with their genetic (left map) and corresponding physical positions on the XRQ genome (right map). **(B)** Enlarged view of the physical map showing the localization of *Pl_*23_HIS33*_*, *Pl_*23_INTER35*_*, and *Pl_*24_HAS62*_* genes. The chromosomal region where *Pl_ARG_* was located (Wieckhorst et al., 2010) is indicated in purple on the physical map. The polymorphic SNP markers of the sunflower AXIOM^®^ array denominated AX-xxxxxxxxx are indicated with their physical positions on the sunflower genomic sequence. **(C)** Enlarged view of the physical map showing the localization of *Pl_*25_HAS40*_* gene. *Pl_*14*_, Pl_*13*_*, and *Pl_*16*_* resistance genes were localized according to published linked genetic markers (Mulpuri et al., 2009; Bachlava et al., 2011; Liu et al., 2012).

The resistances in HIS33, INTER35, HAS62, and HAS40 were mapped on chromosome 1 (Figure 2A). The first three co-localize with markers for *Pl_ARG_* (Wieckhorst et al., 2010) in an area representing 20 to 30 Mb on the reference genome sequence and all overlap (Figure 2B). In contrast, the resistance of HAS40 appears situated in the region carrying *Pl_*13*_, Pl_*14*_*, and *Pl_*16*_* (Liu et al., 2012) but differs from these genes since it is effective against more pathotypes (Figure 2C). Comparison with the *Pl* genes previously mapped was rather difficult as there are differences in marker and gene orders in the genotypes that were used for the crosses. The gene in HAS40 mapped above the SNP marker HT636 and so appears different from *Pl_*13*_* and *Pl_*16*_*, which were mapped below this marker (Mulpuri et al., 2009; Liu et al., 2012) (Figure 2C). *Pl_*14*_* also mapped above this marker but the RGCs identified in this region by Bachlava et al. (2011) could not be used in the present study because they mapped by blastn at different locations including on different chromosomes of the reference genome, with high e-values (<10^-100^). The resistance of HAS40 was quite precisely mapped on the genome sequence in a region of 75 Kb, but surprisingly corresponding to a large genetic region of 16.8 cM. This genomic region does not appear to include any RGC in XRQ, where there may have been a deletion.

#### One Resistance Maps to Chromosome 2

**FIGURE 3:**
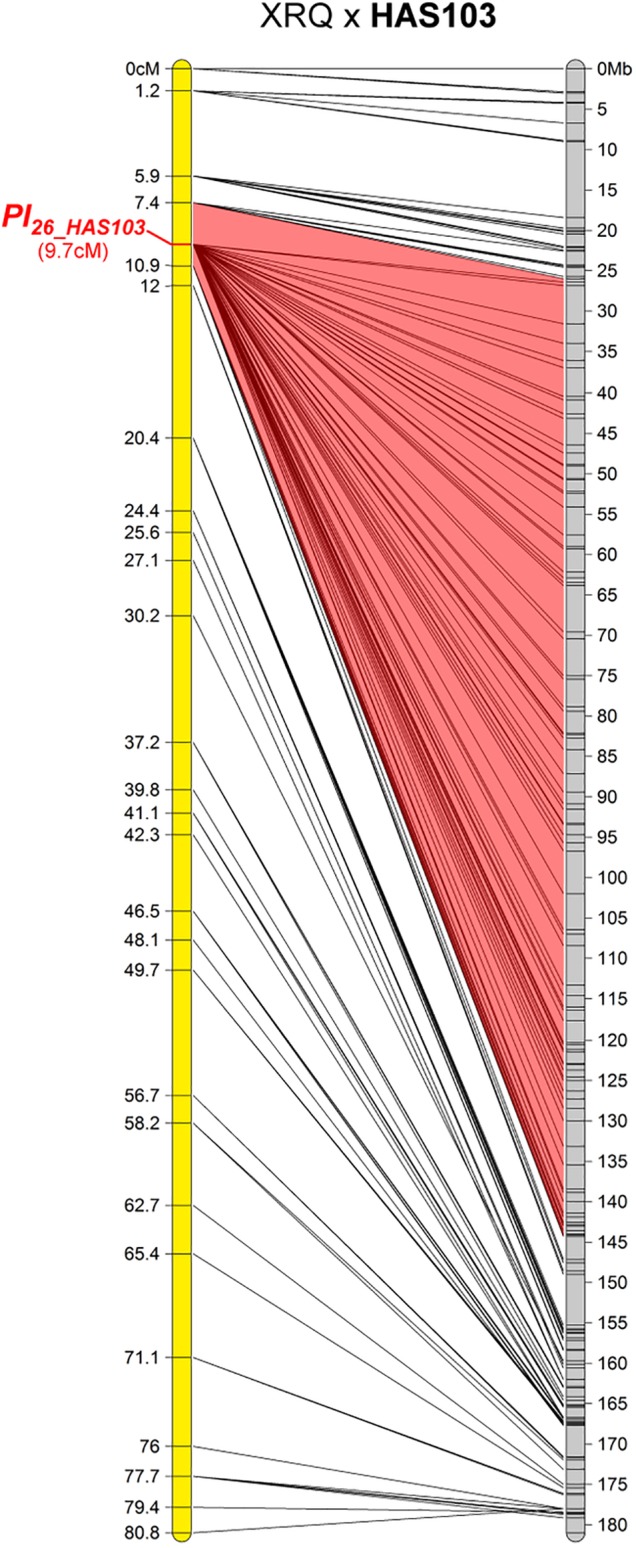
One downy mildew resistance maps to chromosome 2 of sunflower. Genetic (yellow) and physical (gray) map of the *Pl_*26_HAS103*_* resistance gene on chromosome 2. The cross used to map the resistance is indicated on top of the map, with the resistant parent in bold. Only the non-redundant SNP markers are indicated with their genetic (left map) and corresponding physical positions on the XRQ genome (right map).

The resistance of HAS103, a wild *H. annuus* from Kansas, mapped on chromosome 2 (Figure 3). Although on the genetic map the gene appears to be located in only 3.5 cM, on the physical map it is somewhere in 114 Mb. This appears to be related to a lack of recombination on a large part of this chromosome. It is therefore not possible to conclude whether this gene colocalizes with markers for *Pl_*18*_* also on chromosome 2 but coming from *H. argophyllus* (Qi et al., 2016a). HAS103 showed HR with PhRXLR-C01 (Figure 1), its resistance would be distinguished from that of *Pl_*18*_*, if lines containing this gene did not respond to the effector.

#### Four Resistances Map to Chromosome 4

**FIGURE 4:**
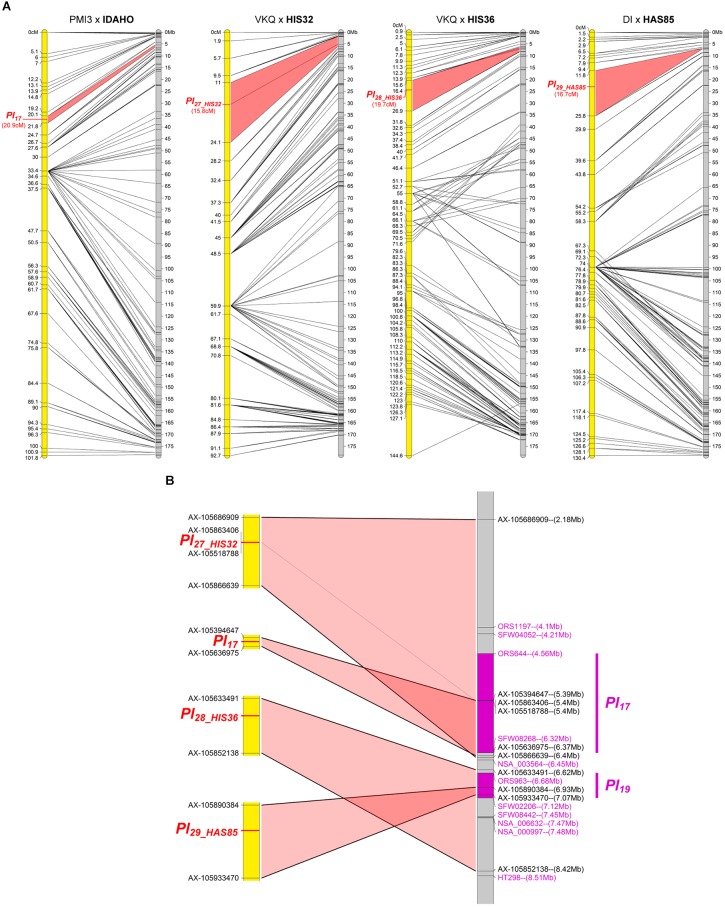
Three downy mildew resistances map to chromosome 4 of sunflower, close to *Pl_*17*_.* **(A)** Genetic (yellow) and physical (gray) maps of the *Pl_*17*_*, *Pl_*27_HIS32*_*, *Pl_*28_HIS36*_*, and *Pl_*29_HAS85*_*, resistance genes on chromosome 4. The cross used to map the resistance is indicated on top of each map, with the resistant parent in bold. Only the non-redundant SNP markers are indicated with their genetic (left map) and corresponding physical positions on the XRQ genome (right map). **(B)** Enlarged view of the physical map showing the localization of *Pl_*27*_*HIS32*_*, *Pl_*28*_*HIS36*_*, and *Pl_*29_HAS85*_* genes. *Pl_*17*_* and *Pl_*19*_* resistance genes were localized according to published linked genetic markers (Qi et al., 2015; Zhang et al., 2017). The polymorphic SNP markers of the sunflower AXIOM^®^ array denominated AX-xxxxxxxxx are indicated with their physical positions on the sunflower XRQ genomic sequence.

The resistances in IDAHO, HIS32, HIS36, and HAS85 mapped in a small region at one end of chromosome 4 (Figure 4A). While those of HIS32 and IDAHO overlap *Pl_*17*_*, those in HIS36 and HAS85 mapped in a distinct region with markers reported to be linked to *Pl_*19*_* (Figure 4B). The resistance of IDAHO, derived from the same *H. annuus* origin as HA458 carrying *Pl_*17*_* and conferring resistance to the same 16 pathotypes, mapped precisely in 1 Mb in part of the area of this gene, so we concluded that it was controlled by the same gene (Qi et al., 2015). In contrast since the resistances of HIS32 and HIS36, both from the *H. tomentosus* breeding pool, mapped to non-overlapping genomic regions of the reference genome, they must, at present, be considered as controlled by different genes. On the physical map they are placed in regions covering *Pl_*17*_* and *Pl_*19*_* respectively but also some distances outside the area of these genes (Figure 4B). The resistance of HAS85 was mapped very close to markers for *Pl_*19*_* (Zhang et al., 2017) and it differs from the three others, IDAHO, HIS32 and HIS36, since it is the only one of the four to respond by a hypersensitive response to the *P. halstedii* RXLR effector PhRXLR-C01 transiently over-expressed (Figure 1 and Table 4).

**Table 4:** Hypersensitive reactions (HR) with *P. halstedii* PhRXLR-C01 effector and resistance gene names.

Chromosome	Sunflower genotype	HR with *P. halstedii* PhRXLR-C01 effector	Origin difference from co-localized genes	*Pl* gene name
1	HIS33	No HR	From *H. resinosus*, *Pl_ARG_* from *H. argophyllus*	*Pl_*23_HIS33*_*
1	INTER35	No HR	From *H. resinosus, Pl_ARG_* from *H. argophyllus*	*Pl_*23_INTER35*_*
1	HAS62	No HR	From wild *H. annuus* Utah, *Pl_ARG_* from *H. argophyllus*	*Pl_*24_HAS62*_*
1	HAS40	No HR	From wild *H. annuus* Texas, *Pl_*13*_, Pl_*14*_, Pl_*16*_* from cultivated sunflower, HAR4, HAR5	*Pl_*25_HAS40*_*
2	HAS103	HR	From wild *H. annuus* Kansas *Pl_*18*_* from *H. argophyllus*	*Pl_*26_HAS103*_*
4	IDAHO	No HR		*Pl_*17*_*
4	HIS32	No HR	From *H. tomentosus, Pl_*17*_* from wild *H. annuus* Idaho	*Pl_*27_HIS32*_*
4	HIS36	No HR	From *H. tomentosus, Pl_*19*_* from wild *H. annuus* Texas	*Pl_*28_HIS36*_*
4	HAS85	HR	From wild *H. annuus* Wyoming, *Pl_*19*_* from wild *H. annuus* Texas	*Pl_*29_HAS85*_*
11	HAS6	HR		*Pl_*30_HAS6*_*
13	HAS42	No HR	From wild *H. annuus* Texas	*Pl_*31_HAS42*_*
13	HAS54	No HR	From wild *H. annuus* Oklahoma	*Pl_*32_HAS54*_*

#### One New Resistance Maps to Chromosome 11

**FIGURE 5:**
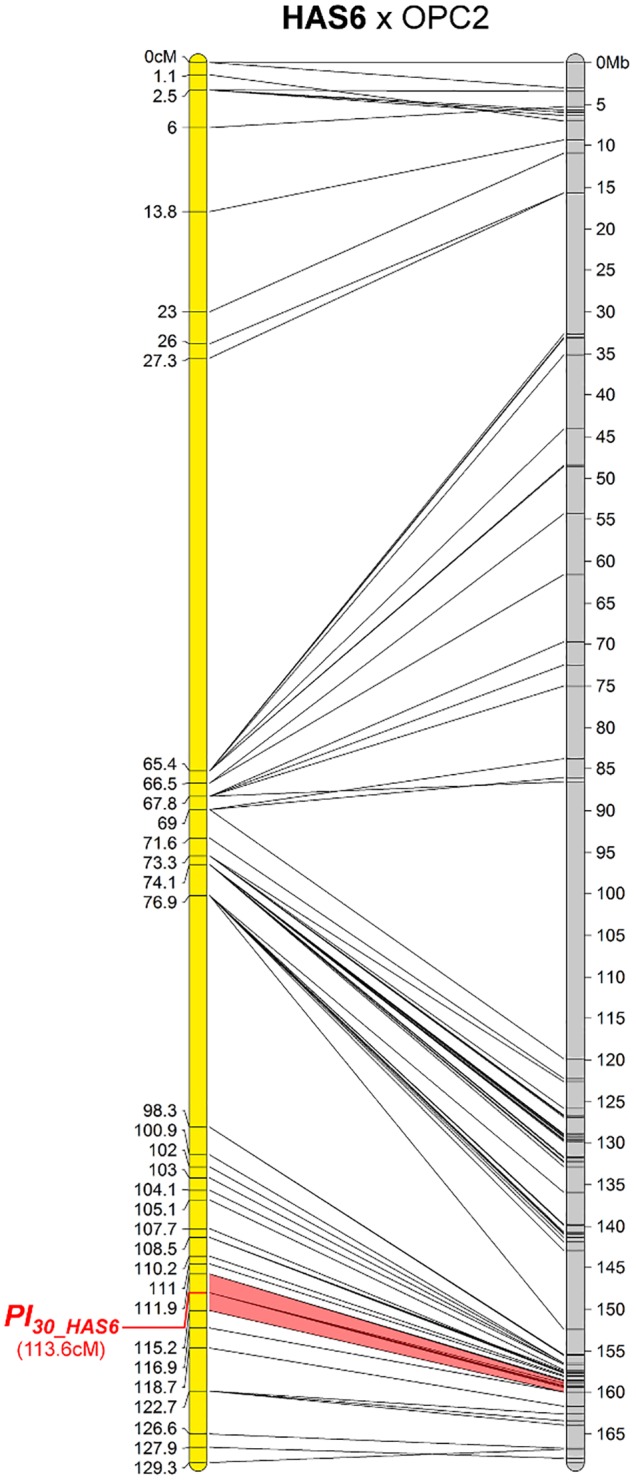
The downy mildew resistance of HAS6 is the first to be localized on chromosome 11 of sunflower. Genetic (yellow) and physical (gray) map of the *Pl_*30_HAS6*_* resistance gene on chromosome 11. The cross used to map the resistance is indicated on top of the map, with the resistant parent in bold. Only the non-redundant SNP markers are indicated with their genetic (left map) and corresponding physical positions on the XRQ genome (right map).

The downy mildew resistance of HAS6 was the first to be mapped on chromosome 11, in a short region spanning 1.3 Mb (Figure 5). This resistance also shows HR with PhRXLR-C01 (Figure 1 and Table 4).

#### Two Resistances Map to Chromosome 13

**FIGURE 6:**
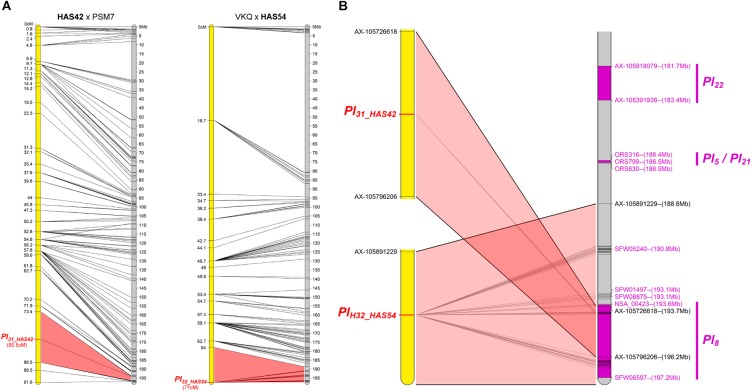
Two new downy mildew resistances map to chromosome 13 of sunflower, close to *Pl8.* **(A)** Genetic (yellow) and physical (gray) maps of the *Pl_*31_HAS42*_* and *Pl_*32_HAS54*_* resistance genes on chromosome 13. The cross used to map the resistance is indicated on top of each map, with the resistant parent in bold. Only the non-redundant SNP markers are indicated with their genetic (left map) and corresponding physical positions on the XRQ genome (right map). **(B)** Enlarged view of the physical map showing the localization of *Pl_*31_HAS42*_* and *Pl_*32_HAS54*_* genes. *Pl_*22*_, Pl_*5*_, Pl_*21*_*, and *Pl_*8*_* resistance genes were localized according to published linked genetic markers (Vincourt et al., 2012; Qi et al., 2017). The polymorphic SNP markers of the sunflower AXIOM^®^ array denominated AX-xxxxxxxxx are indicated with their physical positions on the sunflower XRQ genomic sequence.

The resistances of HAS42 and HAS54 both mapped in a small area on chromosome 13 where *Pl_*5*_, Pl_*8*_, Pl_*21*_*, and *Pl_*22*_* had already been mapped, closest to *Pl_*8*_* (Figure 6). However, they differ from all these genes, either by their reaction to certain downy mildew pathotypes (*Pl_*5*_, Pl_*21*_*, and *Pl_*22*_)* or by their resistance phenotypes (*Pl_*8*_* always shows RII type reactions whereas they show R) (Gascuel et al., 2015).

We positioned these *Pl* genes on the physical map of chromosome 13 according to their linked published markers. Radwan et al. (2003), located *Pl_*5*_* 2 cM and *Pl_*8*_* 4.5 cM from marker NTIR7H3 using two different populations. However, recombination in this area appears to vary between crosses, Qi et al. (2017) reported 5 cM between, ORS316 and *Pl_*8*_* in one cross and 1.2 cM in another. Taking into consideration mapping of *Pl_*5*_* and *Pl_*21*_* below ORS316 but above ORS630 by Vincourt et al. (2012), we propose the map order in Figure 6B. *Pl_*22*_* is above ORS316, with *Pl_*5*_* and *Pl_*21*_* between the two SSR markers and then *Pl_*8*_*, and the resistance genes in HAS42 and HAS54 in the same zone, covering 8 Mb (Figure 6B). Bachlava et al. (2011) reported at least 14 RGC in the region of *Pl_*8*_*, so it is perhaps not surprising that many *Pl* genes map in this area.

Using the AXIOM^®^ genotyping array, the physical mapping of 11 of the 12 resistance genes was consistent with the conclusions of the allelism tests performed initially and the only resistance (HAS40) that could not be localized by phenotypic studies was mapped successfully with the array on chromosome 1.

## Discussion

The results presented here describe mapping, on the sunflower reference genome (Badouin et al., 2017), using a high-throughput mapping SNP AXIOM^®^ array, of 12 sources of resistance effective against at least 16 downy mildew pathotypes. The results suggest that these sources may be considered as providing new resistances carried by 10 novel resistance genes.

### Phenotyping

Before the availability of molecular analyses, determination of linkage or independence of downy mildew resistance genes depended on resistance tests on seedlings with adequate pathotypes. The present results show that a large number of crosses and progenies must be tested for analyses of segregations to permit decisions as to whether *Pl* genes are in one or different clusters. Seventy seven percent of tests reported here did not differ from the Mendelian segregations 3R: 1S, 7R:1S or no segregation. Of those with significant X^2^, some were probably due to the wide use of the highly aggressive pathotype 710, which, in breeding programs, has occasionally been found to give symptoms in plants containing resistance genes but with background genotypes that are unfavorable for expression of resistance. Concerning parental lines, COQ (HA458) and HAS40 showed the highest proportion of “abnormal” segregations (6/13 and 8/17 respectively). For COQ, there was an excess of susceptible plants in five tests out of the six, and it may be suggested that this is due to background susceptibility to pathotype 710. In contrast, for HAS40, five of the eight “abnormal” segregations showed an excess of resistant plants. In three cases they were in crosses with lines (PAA1, HIS33, and HAS62) that were confirmed by the SNP genotyping to have resistance genes on the same region of chromosome 1. These segregations thus probably corresponded to loose linkage between the genes, which could not be estimated precisely with the numbers of plants phenotyped for their downy mildew resistance. It was concluded that phenotyping by resistance tests provided first indications of the genetics of the downy mildew resistances studied but that molecular studies were necessary to confirm distinction and precise location.

### Mapping With SNPs on a Reference Sunflower Genome

The AXIOM^®^ array used for genotyping has theoretically 49,449 SNP. Over the 12 crosses, we found an average of 5953 high-quality polymorphic SNPs, between a minimum of 4138 for HAS40 and a maximum of 8548 for HIS33 (i.e., 17% of the total SNPs present on the array). Although some physical information was lost according to the cross, there were always sufficient markers to map correctly the resistances in regions of 10s of Mb, and sometimes in 10s of kb (HAS40). This was quite surprising since HAS40 had a small number of polymorphic markers on the chromosome concerned (221). However, the F3 population used for mapping was large (70 plants) making it possible to take account of a sufficient number of recombination events. In addition, the segregation ratios observed for resistance were close to theoretical values, suggesting that mapping population size and phenotyping quality are important criteria for precise fine mapping. On the contrary, the cross involving HAS103 had 50% more polymorphic markers on the chromosome concerned, but the mapping population was smaller (45) and segregation for resistance not optimal. Together with a lack of recombination events on chromosome 2 in the HAS103 x XRQ population, the result is poor physical mapping.

Supplementary Figure 1, plotting size of intervals in which resistances were mapped against numbers of markers polymorphic between parental lines on the chromosome concerned, suggests that there could be a relation between polymorphism, a measure of distance between lines, and frequency of recombination. In other words, high levels of polymorphism would disfavor recombination events at the DNA level in segregating progenies, rendering fine mapping more difficult. High levels of polymorphism have already been observed when the parental lines of crosses used to map resistance were of very different origins, with one parent from wild *H. annuus* ecotypes or from different *Helianthus* species. In the first report mapping *Pl_ARG_*, chromosome 1 appeared much shorter in a cross involving *H. argophyllus* and cultivated sunflower (Dussle et al., 2004) than in crosses between two cultivated sunflower lines (Wieckhorst et al., 2010). Moreover, it has recently been shown in rice that regions with high levels of polymorphism, in some cases associated with clusters of resistance genes, display reduced crossover frequencies (Mieulet et al., 2018); illustrating a negative correlation between genome divergence of the parental lines and recombination rate.

In addition, this work permits, for the first time, comparisons between genetic and physical maps in sunflower, for 5 of the total 17 chromosomes and highlights several interesting features. First, we observed the presence of differences between the two maps, particularly concerning chromosomes 1, 11, and 13. On the physical map of the reference genome, there appear to be some large genetic regions (10s of cM) devoid of SNPs, whereas the SNPs selected for the study provide relatively regular chromosome coverage. Thus, in the populations studied, the recombination frequency may be high in these regions compared with the reference genome. Alternatively, there may have been deletions in the reference genome compared with the genotypes used for mapping. Second, we observed inversions between genetic and physical maps, for example on chromosome 4, again suggesting differences between the parental genotypes used to map the resistances and the reference genome. It would be interesting to have the sequence of more than one genome to allow better comparisons with genotypes of various origins and to understand sunflower genome evolution.

### Physical Mapping of New and Published *Pl* Resistances

Mapping and positioning physically on the reference genome made it possible to compare localizations of new downy mildew resistance genes from different genetic backgrounds and also *Pl* genes that were previously genetically mapped in the same regions. The genetic regions defined for formerly mapped *Pl* genes appear to be very large on the physical map, encompassing up to 10 Mb for *Pl_ARG_* and up to 20 Mb for *Pl_*14*_*, both on chromosome 1. Badouin et al. (2017) estimated that 1 Mb on the XRQ sequenced genome contains an average of 20 genes. It is therefore highly probable to find closely linked but different resistance genes in such large regions, and one of the reasons why we propose two new genes, close to *Pl_ARG_* on chromosome 1. However, it should be kept in mind that gene order in some genotypes could be slightly different from that in the reference genotype, making fine mapping difficult.

### Gene Distinction and Nomenclature

Even with the availability of a sunflower genome sequence, all the questions regarding the genetic and molecular architecture of resistance to downy mildew will not be solved in the near future, so it appears of interest to fix a rule to designate genes in sunflower involved in the interaction with *P. halstedii.*

In the past, if two resistance sources had resistance to the same downy mildew pathotypes and showed no segregation in test crosses, they were considered to carry the same gene. For example two of the pathotype differential lines (Gascuel et al., 2015), PM17 (D5) and 803.1 (D6), showed no segregation when crossed with XRQ, carrying *Pl5*, but they showed slight differences in phenotyping reactions to pathotypes: PM17 is susceptible to a Spanish isolate of pathotype 330 to which XRQ is resistant, so it was considered to have *Pl_*5*-_* whereas 803.1 is resistant to the US isolate of 330 to which XRQ is susceptible so its gene was defined as *Pl_*5*+_* (Vear et al., 2008a). If they had been mapped recently, they would probably been given different names. To distinguish resistance sources with closely linked genes by segregation studies would require very large populations and certainty that observations of one or a few susceptible plants were correct. At present, there are many sources that cannot be distinguished by their pathotype reaction, because they are effective against all known pathotypes. Some of them are located in the same genomic regions, but since slightly different markers are polymorphic according to the lines studied, it is difficult to be certain that they are in exactly the same place and to decide whether they are different genes. An example involved in mapping of the resistance of HAS40 concerned *Pl_*13*_, Pl_*14*_* and *Pl_*16*_* that gave initially identical reactions to downy mildew pathotypes_._ These genes were identified in rust resistant pools developed by USDA from Argentinean origins: *Pl_*13*_* in HAR5 (Mulpuri et al., 2009), *Pl_*14*_* in a line derived from HAR4 (Bachlava et al., 2011) and *Pl_*16*_* in HAR4 (Liu et al., 2012). All three genes were mapped to the lower end of chromosome 1, but, surprisingly, and depending on the position of one marker HT636, the two genes from HAR4, *Pl_*14*_* and *Pl_*16*_* appeared to be at different positions, the latter being mapped in the same region as *Pl_*13*_* according to our results. However, recent results confirm that *Pl_*13*_* differs from *Pl_*14*_* and *Pl_*16*_* because pathotype 705, differentiating these resistances, was reported by Garcia-Carneros and Molinero-Ruiz (2017) and all the genes in this cluster may be slightly different, comparable with those on chromosome 13.

Recent studies of presence or absence of host reactions to pathogen effectors provide new evidence that some of the genes situated in the same region are different (Pecrix et al., 2018). In the case of chromosome 1, none of the four sources responded by an HR to the *P. halstedii* effector PhRXLR-C01. However, this test was decisive to distinguish the resistance in HAS85 from *Pl_*17*_* and the genes in the two other origins (HIS32 and HIS36) located in the same region of chromosome 4, since only HAS85 responded by an HR. It would be of interest to test the reaction of a line carrying *Pl_*19*_* to this effector to determine whether the resistance of HAS85 is distinguished from this gene. On chromosome 13, effector reaction also distinguishes *Pl_*22*_* in line PMI3 (HR to PhRXLR-C01, Figure 1) from all the other genes in this region which show no HR.

Nevertheless, for many of the new resistance genes, until their sequences and their possibility of reacting differently to different pathogen genotypes, have been analyzed in detail, it is difficult to know whether all merit new names. Mapped regions vary from 0.07 Mb (about two genes) for the gene in HAS40 to 32 Mb (640 genes) for the other resistances on chromosome 1, in HIS33, INTER35 and HAS62. At this scale, we cannot conclude between close genes or one gene so we have tried to fix some rules for naming the genes identified in the present study.

Firstly, those that map in the same area and come from the same origin are given the same name: IDAHO, whose gene mapped to chromosome 4, was bred from the same origin as HA458 so it was considered that it carries *Pl_*17*_*. For the lines selected from interspecific breeding pools, detailed history is not available and downy mildew reactions were not studied during fixation. With no proof of difference, and the resistances of HIS33 and INTER35 (from *H. resinosus*) both being mapped in the same area on chromosome 1, it appears most logical to consider that only one resistance gene was introgressed. Therefore, we propose to call the gene from *H. resinosus Pl_*23*_*. For the other lines, several resistances map in the same zone as known genes, and others appear to co-localize, but, in all cases they come from different species or from ecotypes collected in different geographical origins. We therefore suggest that they should be given different names, at least for the present (*Pl_*24*_–Pl_*32*_*) and we include in the name the source of the resistance. These are listed in Table 4. They could make a total of 10 additional genes available in breeding for resistance to sunflower downy mildew.

With more than 30 downy mildew resistance genes identified and positioned on the sunflower genome, simple numbering according to order of identification is not helpful to avoid errors. It might be useful to include at least chromosome number in gene names. Of course, international agreement would be necessary, as for the triplet denomination of downy mildew pathotypes (Gulya et al., 1998).

These results confirm that genes controlling resistance to sunflower downy mildew are found in clusters (Radwan et al., 2008). In the literature, many clusters of NBS-LRR corresponding to putative resistance genes were found in several *Pl* regions (Radwan et al., 2003, 2004), comparable with those for *Bremia lactucae* in lettuce (Michelmore and Wong, 2008) and with other pathogens in bean (Meziadi et al., 2016). At least six chromosomes are concerned, *Pl_*30*_*, in HAS6, being the first downy mildew resistance gene reported to map to chromosome 11, although some rust resistance genes have already been mapped on this chromosome (Zhang et al., 2016). *Pl_ARG_* was reported as not clustered (Dimitrijevic and Horn, 2018) but localization of *Pl_*23*_* and *Pl_*24*_* show that this is not the case. The different clusters could in part be duplications as sunflower is considered to have undergone chromosome doubling (Badouin et al., 2017) but the many genes in one cluster could well be sequences that have been imperfectly duplicated or the result of unequal crossing-overs as has been shown for other major resistance genes (Meziadi et al., 2016).

We performed the colinearity studies of the genetic maps using the genome sequence of the cultivated sunflower line XRQ (Badouin et al., 2017). However, although this resource was a major breakthrough for genetic studies in sunflower such as for the mapping of the resistance genes to downy mildew of our study, this reference sequence does not represent the diversity in cultivated sunflower and even less the diversity in wild *Helianthus*. Plant genomes vary considerably, including within the same species. It is now relatively easy to re-sequence genomes and identify SNP polymorphisms by comparing the re-sequencing data with a reference sequence. However, the structural variations (inversion, translocation, deletion, insertion, or copy number variation) that can range from a few nucleotides to 100s of kb, are more difficult to identify. By aligning genetic maps on the reference genome sequence, we identified such polymorphisms which suggest that some resistances, originating from wild sunflowers, could be due to structural variations. In order to facilitate the sequencing of the downy mildew resistance genes or other loci of interest, it would be very useful to produce new tools or new genomic resources in sunflower. For example, from a pan-genome, it would be interesting to develop a genotyping tool based on SNPs identified on this pan-genome in order to identify large structural variations. At a finer scale, making the optical maps of the genomes from 10s of accessions would be a useful resource too. Finally, on an even finer scale, assembling high quality genome sequences of sunflowers and wild *Helianthus* would considerably speed up research in sunflower, which could become a model plant species in many scientific fields (genetics, evolution, floral development, responses to biotic and abiotic stresses...).

The present results suggest that *Helianthus* species, and more particularly wild *H. annuus* are quite rich in broad spectrum downy mildew resistance genes. Use of combinations of those described here should help to provide some reasonably durable resistance, protecting the sunflower crop against changes in downy mildew pathotypes.

## Ethics Statement

Sunflower plant material used in this study was grown from seeds made available by INRA as part of INRA sunflower genetic resources. The lines Idaho, COQ and U8Q were obtained by selfing from USDA sources.

## Author Contributions

YP, FV, and LG conceived the project. FV produced the plant material and carried out phenotyping. CP-S and YP produced the plant material for genotyping. SM designed the 50K-SNP AXIOM^®^ array. YP and LG acquired genotyping data. YP mapped the resistance genes. FV, LG, SM, and YP wrote the manuscript. All authors were involved in revising the manuscript critically and approved it.

## Conflict of Interest Statement

The authors declare that the research was conducted in the absence of any commercial or financial relationships that could be construed as a potential conflict of interest.

## Footnotes

1 http://www.affymetrix.com

2 https://www.heliagene.org/
