# Supplementary material for: Ten Broad Spectrum Resistances to Downy Mildew Physically Mapped on the Sunflower Genome
Source: Front Plant Sci. 2018 Dec 4;9:1780. doi: 10.3389/fpls.2018.01780 (PMC6288771; doi:10.3389/fpls.2018.01780)
Supplement: Supplementary file 1 [file Table_1.DOCX]

Supplementary Material

**Ten Genes for Broad Spectrum Resistance to Downy Mildew Physically Mapped on the Sunflower Genome**

**Yann Pecrix, Charlotte Penouilh-Suzette, Stéphane Muños, Felicity Vear* and Laurence Godiard*.**

*** Correspondence:** Corresponding Authors: [felicity.vear@wanadoo.fr](mailto:felicity.vear@wanadoo.fr) [laurence.godiard@inra.fr](mailto:laurence.godiard@inra.fr)

**Supplementary Figure 1. Relationship between the physical size of intervals containing each resistance gene and the number of markers polymorphic between parental lines on the chromosome concerned.**

The size of intervals are indicated in bp, and transformed by logarithm in base 2 (log2) and the number of markers polymorphic between parental lines is divided by the size in Mb of the chromosome concerned.


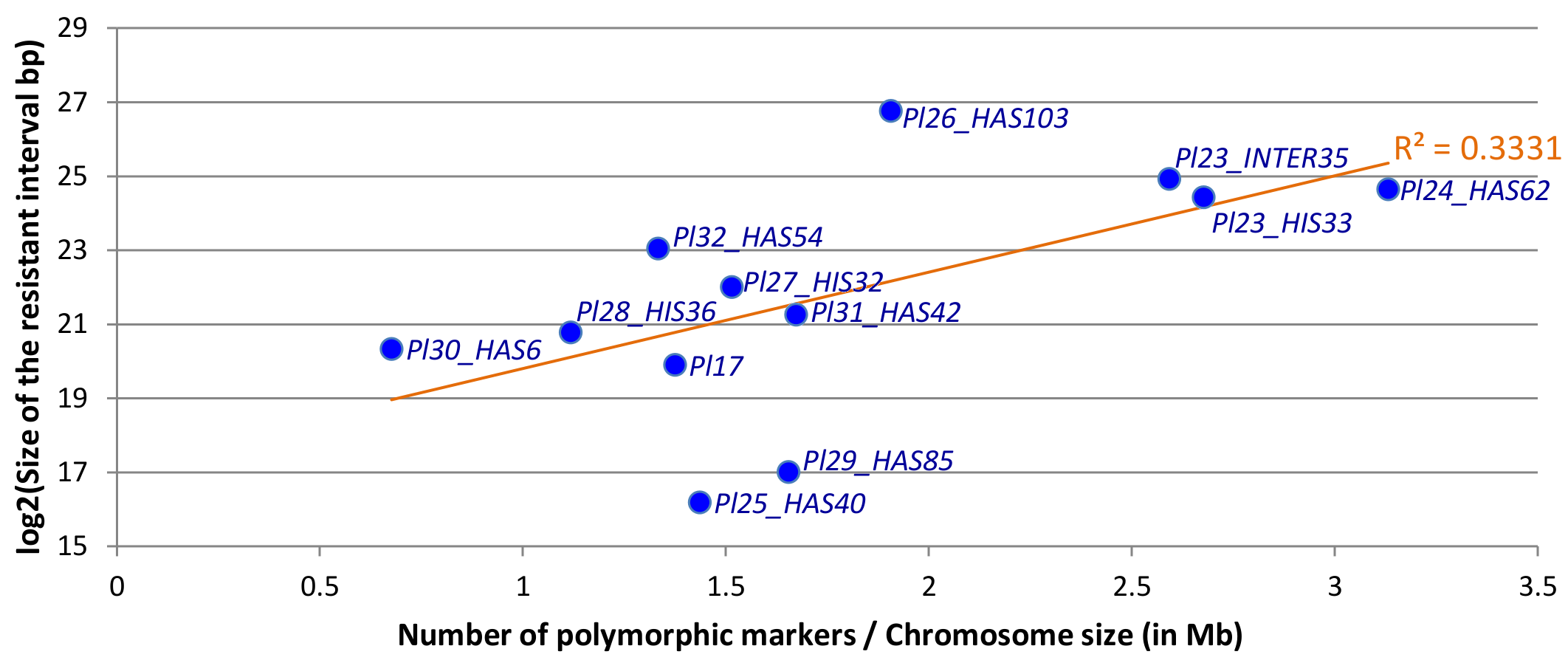


**Supplementary Tables** provided as an excel file **“**Supplementary Tables_Pecrix.xlsx”

**Supplementary Table 1. Summary of test cross progenies performed between resistance sources.**

**Supplementary Table 2.** **Downy mildew resistance segregations of test cross progenies** (susceptible x F1 between resistance sources). Segregations in red do not agree with the hypotheses tested.

| **Supplementary Table 3. F3 progenies genotypes and numbers of markers used.** |
| --- |
| RR: homozygous resistant; SEG: progeny segregating for resistance; SS: homozygous susceptible. |
| **Supplementary Table 4. Details of markers co-localizing with downy mildew resistances.** No markers co-localized with resistance in lines HAS40, HIS36, IDAHO and HAS85. |
